# Benign tremulous Parkinsonism: a unique entity or another facet of Parkinson’s disease?

**DOI:** 10.1186/s40035-016-0057-1

**Published:** 2016-05-20

**Authors:** Wissam Deeb, Wei Hu, Leonardo Almeida, Addie Patterson, Daniel Martinez-Ramirez, Aparna Wagle Shukla

**Affiliations:** Department of Neurology, University of Florida Health - College of Medicine, Center for Movement Disorders and Neurorestoration, Gainesville, FL USA

## Abstract

Benign tremulous parkinsonism (BTP) is characterized by a prominent tremor that occurs both at rest and with action in conjunction with other mild features of parkinsonism. The progression of symptoms is typically slow and there is often a positive family history. Although BTP is included within the phenotypic spectrum of Parkinsonism its exact relationship with idiopathic Parkinson’s disease remains unclear. Treatment of BTP is challenging especially considering the poor response to levodopa, therefore surgical therapies such as deep brain stimulation surgery are sought for treatment of these tremors. In this review, we will summarize the clinical features, diagnosis, neuropathology and treatment for BTP.

## Background

Tremor is a common symptom seen in movement disorders clinical practice [1, 19]. The most common cause of tremors is essential tremor and the other important differential is idiopathic Parkinson’s disease (PD)[2–5]. The basic difference between the arm tremors seen in these two conditions is that essential tremor is characterized by postural and action tremor whereas PD tremor is mostly a resting tremor [11]. Benign tremulous parkinsonism was first characterized extensively in cohort of 16 patients followed at Mayo Clinic who shared similar phenotype and exhibited a unique clinical course. These patients had a pronounced asymmetric resting tremor in conjunction with mild to moderate postural and action tremor and mild parkinsonian signs. The tremors did not respond to alcohol. Although this constellation of features was tempting to be categorized as PD, the clinicians at Mayo Clinic observed unique differences. The progression of symptoms was notably slow, there was a significant family history and unlike typical PD, there were fewer nonmotor features. Prior to this report, there were only few cases briefly mentioned in the literature. The authors referred to this condition as benign tremulous Parkinsonism (BTP). Since then a debate has surfaced whether BTP represents a unique disorder or the clinicians are facing a variant within the spectrum of tremor-predominant idiopathic PD [10, 42].

**Table 1:** Clinical Characteristics of patients with Benign Tremulous Parkinsonism

Study	Sex (M/F)	Mean Age of onset (range)	Initial symptoms	Disease duration (years, range)	Treatment response	Family history
Josephs et al.	10/6	58.5 (42–71)	Unilateral hand rest tremor (13/16)	11.1 (8–25)	No benefit by LD^a^(≥600 mg/d)	10/16
Selikhova et al.	9/7	59 (30–72)	Unilateral hand rest tremor (11/16), rest and postural/action tremor (5/16)	24 (12–50)	Not responsive to LD (9/15), though 6/15 had early benefit	5/15
Leventoglu et al.	16/7	66.6 (50–89)	Unilateral hand rest tremor (18/23)	5	Little improvement to LD (11/18)	4/23

^a^ *LD* Levodopa

According to the Mayo Clinic series [21] which was also later reinforced by other groups [26, 41], BTP is characterized by rest and action tremor at onset that remains persistent as the dominant feature for at least 8 years with only a minimal progression of other parkinsonism signs. The gait disorder essentially comprises of mild stooping or a reduced arm swing instead of classic shuffling gait with progressive deterioration of balance. These patients display a poor and unpredictable response to levodopa therapy. Most of them present at a relatively younger age of onset in the fifth decade and have a family history which suggests a strong underlying genetic etiology. The prognosis is much better compared to idiopathic PD. Postmortem findings indicate fewer pathological changes in the substantia nigra further consolidating the concept that BTP is a distinct entity [41]. Some argue BTP represents one end of the clinical spectrum of PD which is tremor-predominant PD [10]. This subtype typically progresses the slowest amongst the various PD subtypes and the presence of significant motor findings in the beginning may be minimal. In an editorial on study by Selikhova et al., it was recommended that patients with rest and action tremors and minimal Parkinsonism features should be categorized as “monosymptomatic tremor at rest”. Since only some of the patients initially diagnosed as BTP eventually displayed typical pathological features of PD, it was felt that BTP was a misnomer [10] (Table 1).

Whether BTP should truly be considered “benign” is controversial. The term “benign” was proposed due to a slower rate of progression of other Parkinsonism symptoms which could indicate a better prognosis than idiopathic PD. However, there is a suggestion that these patients may also have sudden worsening of other Parkinsonian signs, such as rigidity and gait resulting in significant motor disability [39]. Another example of “benign” movement disorder, is essential tremor which was labeled as “benign” for decades in literature until the most recent years, when gait dysfunction and memory problems in ET became well-known. Considering these additional features, some experts recommend avoiding the term “benign” when describing ET. Similarly, clinicians must also be cautious regarding the usage of the term “benign” in the context of BTP until further longitudinal studies can better establish or refute such an assumption. Follow-up studies should focus on the rate of progression, and the co-occurrence of non-motor symptoms.

### Pathological findings in BTP

There is scant knowledge on the neuropathology of BTP. In one study, Selikhova M. et al. compared brain pathology specimens of 15 BTP cases with age and disease duration-matched PD controls. They found that most BTP patients had lesser neuronal loss in the ventrolateral portion of the substantia nigra than their PD controls. Similar to PD, BTP cases had Lewy bodies in the substantia nigra and other brainstem structures. These findings suggest there is a slower nigral cell degeneration which correlates with relatively stable Parkinsonism observed throughout the clinical course of BTP. It is noteworthy that tremor predominant subtype of PD also has lesser neuronal loss in locus coeruleus (LC) and in medial and lateral parts of substantia nigra [20]. Therefore, it will be worthwhile to conduct a pathology study that could identify the key differences between BTP and tremor predominant PD.

### Genetic aspects

A striking finding to note with BTP is the positive family history of tremor identified in nearly 31 to 63 % of patients [21, 41], in contrast to only 7 to 16 % of idiopathic PD patients [12, 32, 34, 35, 38]. These findings are in contrast to a lower prevalence of family history in idiopathic PD (<10 %) and highlight an underlying genetic etiology to play a substantial role.

The genetic underpinnings of BTP albeit intriguing, only few cases have shown positive gene tests. LRRK2 mutation is responsible for about 1 % of sporadic PD patients and 4 % of hereditary PD cases. Clarimon J. et al. in a series of patients who had clinical characteristics similar to BTP, found new LRRK2 (V2390M) mutation. Although authors allude to clinical similarities between their patients and the BTP case series from Mayo, the authors refer to the patients as tremor dominant parkinsonism [9]. Rizzo et al. [37] described another patient with Dardarin mutation who had clinical presentation consistent with BTP. In addition, compound heterozygous parkin gene mutation was identified in one patient [41]. Both LRRK2 mutations and parkin gene have been linked to various heterogeneous phenotypes in idiopathic PD [7, 16]. Considering the presence of significant family history, further studies are required to identify the underlying genetic aspect of BTP [29, 30].

### Clinical features

As reported in multiple case series [21, 26, 41], the presenting symptom in BTP is a moderate asymmetric upper limb tremor [21], with majority presenting with a combination of rest and postural/action tremors [41]. These tremors tend to only slowly worsen over time, and typically do not respond to alcohol [21]. In the early course of BTP, only mild features of Parkinsonism such as bradykinesia, rigidity, stooped posture and a reduced arm swing were observed [21, 41]. Interestingly, these Parkinsonian signs remain relatively unchanged for many years. Only few patients develop mild non-motor symptoms including dysautonomia, constipation, erectile dysfunction, and bladder dysfunction [21]. There is a male predominance and the age of onset is relatively younger ranging from 58.5 to 66.6 years [21, 26, 41].

### Diagnosis

It is important to differentiate BTP from other similar tremor syndromes [36], including the commonly seen tremor-predominant PD and essential tremor. Each alternative tremor syndromes has specific features which should be given due consideration when making the diagnosis.

1. Mono-symptomatic resting tremor, identified as a pure or predominant rest tremor: These patients however do not have other Parkinsonism symptoms and signs [11].
2. Essential tremor: BTP tremors are postural +/− kinetic and there is a strong family history. However unlike the tremor of BTP, use of alcohol attenuates tremors and there are no accompanying features of parkinsonism [22].
3. Isolated tremor and subtle Parkinsonian signs relevant to aging [25]: The parkinsonian features manifest at an older age which is different from BTP in which the mean age of onset is usually less than 60 years.
4. PD initially manifesting as essential tremor: These patients [6, 15], are characterized by a postural tremor or head tremor who develop parkinsonian symptoms later in life. Their clinical features and disease course are consistent with typical idiopathic PD.
5. Combined essential tremor with PD [24]: In this syndrome the rate of progression for PD follows the typical course unlike patients with BTP.
6. “Tremor-predominant PD”: This term encompasses a specific phenotype of PD to distinguish patients who have prominent tremors as the main clinical presentation from those who have akinetic-rigid type or those who can be categorized into postural instability-gait disturbance phenotype. The term has a broader connotation and does not necessarily imply presence of other key features of BTP including remarkable family history, and a slow course of progression.

BTP is relatively new, therefore lacks clinical validity and specific diagnostic criteria. In the early stages, it maybe difficult to clinically differentiate BTP from typical idiopathic PD. If there is dopamine depletion in the brain, the patient is likely to suffer from PD [28]. The clinicians should consider BTP if the patients present with prominent rest tremor in conjunction with postural and action tremors. BTP becomes very likely if tremor remains the major sign/symptom with mild and stable Parkinsonism despite several years of follow-up. The other clinical clues include the absence of a satisfactory response to levodopa therapy and a positive family history. A Dopamine transporter Single-photon emission computed tomography (DAT SPECT) test which is sensitive and specific for diagnosis of degeneration related parkinsonism has not been reported in BTP, may be helpful for diagnosis. If BTP is distinct from tremor predominant PD, DAT SPECT is expected to be normal [36].

### Treatment

BTP patients have usually shown refractoriness to levodopa therapy, particularly in the early disease stages, despite reaching average doses of 900 mg/day [21, 40]. Selikhova et al. [41] found that in a series of 12 patients, six patients had a transient early response to high doses of levodopa (400–1250 mg/day), and over half of the patients did not have a satisfactory response to dopaminergic therapy [41]. Interestingly, substantial levodopa complications including dyskinesias have not been described in these patients. The effect of medications other than levodopa, particularly the neuroprotective agents [8, 31] and anti-tremor medications, on the course and tremor symptoms in these patients require further studies.

Over the past decade, a large body of evidence has established the application of deep brain stimulation (DBS) surgery in the management of medication refractory tremors for PD [13, 14, 18, 23, 27] and essential tremor [17, 33]. A review of 15 BTP patients who received DBS showed significant improvement of symptoms [40]. Of these, 12 patients received ventral intermediate (VIM) nucleus of the thalamus (8 patients with unilateral and 4 with bilateral leads). The remaining three patients received bilateral subthalamic nucleus (STN) stimulation. All of these patients received a mean levodopa challenge dose of 900 mg/d (300 mg 3 times daily), with about half of them showing an initial but suboptimal response, whereas the other half had no benefit with levodopa use. Overall there was a satisfactory response to either VIM or STN stimulation, with seven patients being tremor-free and six patients remaining with minimal residual tremor at their last follow-up visit. Upon reviewing these impressive benefits, it appears BTP patients would be suitable for DBS at earlier stages of disease. VIM could be an appropriate target as tremors are the predominant feature and other Parkinsonian signs are mild. The current available data, is not adequate to establish practice parameters for DBS which could be accomplished through future well-powered studies.

## Conclusion

BTP is clinically characterized by asymmetric tremors at onset, tremors present at rest, postural and action tremors and they remain as the prominent clinical feature for many years. These tremors donot respond to alcohol. Tremors are accompanied by other mild features of parkinsonism which do not progress at typical rates. Unlike typical PD, there family history is significant and the nonmotor features are not that frequent. Autopsy studies have found fewer pathological changes in the substantia nigra. Whether BTP is a distinct clinical entity or is a subtype of PD is currently debatable. Treatment of BTP is challenging, response to levodopa is poor even when tried at high doses. Athough small scale studies have shown bilateral DBS of VIM and STN to exhibit excellent outcomes, prospective studies with larger sample sizes, and randomized target selection, are required to determine the long-term efficacy and durability [40].
